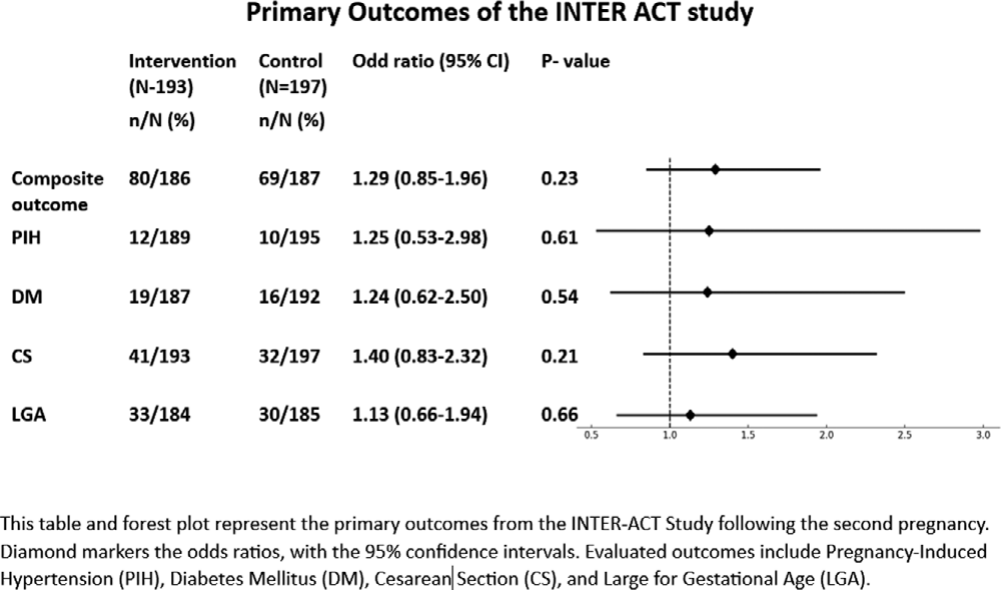# Oral Plenary Session 1: Thursday, January 30, 2025 8 AM – 10 AM

**DOI:** 10.1002/pmf2.12002

**Published:** 2025-01-05

## 1 | Antenatal Corticosteroid in Twin Pregnancy at Risk of Late Preterm Birth: A Randomized Controlled Trial

Seung Mi Lee^1^; Hyun Soo Park^2^; Soo Ran Choi^3^; Jeesun Lee^1^; Hyun Ji Kim^4^; Jee Yoon Park^4^; Kyung Joon Oh^4^; Geum Joon Cho^5^; Min‐Jeong Oh^5^; Jin Hoon Chung^6^; Sun Min Kim^7^; Byoung Jae Kim^8^; Suk Young Kim^9^; Subeen Hong^10^; Young Mi Jung^5^; Se Jin Lee^11^; Ji Su Seong^12^; Haemin Kim^13^; Sohee Oh^8^; Joongyub Lee^1^; Ji Hoi Kim^1^; Hee Young Cho^14^; Chan‐Wook Park^1^; Joong Shin Park^1^; Jong Kwan Jun^15^

*^1^Seoul National University College of Medicine, Seoul, Seoul‐t'ukpyolsi; ^2^Dongguk University Ilsan Hospital, Goyang, Kyonggi‐do; ^3^Inha University Hospital, Inha University College of Medicine, Incheon, Inch'on‐jikhalsi; ^4^Seoul National University Bundang Hospital, Seoul National University College of Medicine, Bundang, Kyonggi‐do; ^5^Korea University College of Medicine, Seoul, Seoul‐t'ukpyolsi; ^6^University of Ulsan College of Medicine, Asan Medical Center, Seoul, Seoul‐t'ukpyolsi; ^7^101 Daehak‐ro, Jongno‐gu, Seoul, Seoul‐t'ukpyolsi; ^8^Seoul Metropolitan Government Seoul National University Boramae Medical Center, Seoul, Seoul‐t'ukpyolsi; ^9^College of Medicine, Gachon University, Incheon, Inch'on‐jikhalsi; ^10^Seoul St. Mary's Hospital, College of Medicine, The Catholic University of Korea, Seoul, Seoul‐t'ukpyolsi; ^11^Kangwon National University Hospital, School of Medicine, Kangwon National University, Chuncheon, Kangwon‐do; ^12^Chung‐Ang University Gwang‐Myeong Hospital, Chung‐Ang University College of Medicine, Gwang‐Myeong, Kyonggi‐do; ^13^Kyungpook National University Chilgok Hospital, Kyungpook National University, School of Medicine, Daegu, Ch'ungch'ong‐bukto; ^14^Seoul National University, Seoul, Seoul‐t'ukpyolsi; ^15^Ewha Womans University College of Medicine, Seoul, Seoul‐t'ukpyolsi*

8:00 AM ‐ 8:15 AM

**Objective**: Recently, guidelines recommend corticosteroid injection in singleton pregnancy at risk of late preterm birth (34 ∼36+6 weeks). However, the effectiveness of antenatal corticosteroid in twin pregnant women at risk of late preterm birth has not been evaluated, and there is a paucity of guideline in this population. In the current study, we evaluated if antenatal betamethasone administration reduces the risk of neonatal respiratory morbidity in late preterm twin neonates.

**Study Design**: In this multicenter randomized trial, we enrolled twin pregnant women at 34+0 to 36+5 weeks of gestation who were at risk of late preterm birth. The participants received betamethasone or placebo after randomization of 1:1 ratio. Primary outcome was severe neonatal respiratory morbidity, including at least one of the followings: the use of continuous positive airway pressure (CPAP) or high‐flow nasal cannula for 12 hours or more, supplementary oxygen administration with a fraction of oxygen of at least 0.3 for 24 hours or more, mechanical ventilation, extracorporeal membranes oxygenation, or perinatal death within 72 hours after birth. The secondary outcome was other neonatal morbidities. 

**Results**: Among 1620 neonates, the primary outcome occurred in 99 neonates with lower risk in the betamethasone group than in placebo group (39/818 [4.8%] vs. 60/802 [7.5%], relative risk (RR) 0.64 [95% CI, 0.42‐0.98]) Other respirator morbidities were also lower in the betamethasone group, such as CPAP use for 2 hours or more (RR 0.58 [95% CI 0.35∼0.95]) and transient tachypnea of the newborn (RR 0.47 [95% CI 0.25∼0.89]). The risk of neonatal hypoglycemia was increased in the betamethasone group (RR 1.33 [95% CI 1.01∼1.75]), but the risk of neonatal sepsis or maternal chorioamnionitis were not different between the two groups of cases.

**Conclusion**: The antenatal betamethasone administration in twin pregnant women at risk of late preterm birth significantly reduced the risk of neonatal respiratory morbidity.

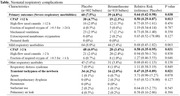

## 2 | The Frequency and Severity of Complications after Previable PROM in Texas following SB8

Danna Ghafir^1^; Emily Fahl^1^; Nancy Ukoh^1^; Han‐Yang M. Chen^1^; Sean C. Blackwell^1^; Julie Gutierrez^2^; Irene A. Stafford^1^

*^1^McGovern Medical School at UTHealth Houston, Houston, TX; ^2^McGovern Medical School at The University of Texas Health Science Center at Houston (UTHealth), Houston, TX*

8:15 AM ‐ 8:30 AM

**Objective**: Robust contemporary data concerning previable PROM are limited. The impact of changes in state abortion laws on maternal outcomes also remains unknown. We sought to compare outcomes of pregnant individuals who develop previable PROM at our center before and after the implementation of Texas Senate Bill 8 (SB8).

**Study Design**: This is a retrospective cohort study across three tertiary care hospitals from January 1, 2018‐March 31, 2023. ICD‐10 codes were used to identify individuals with previable PROM. Chart review was done by trained research staff. Individuals with singleton or twin gestions and clinical diagnosis of PROM < 22 weeks were studied, excluding those with IUFD and/or active PTL. We compared clinical characteristics and outcomes of individuals before and after the implementation of SB8 on September 1, 2021. Our primary outcome was composite adverse outcome, which included ICU admission, transfusion, and/or sepsis. Multivariable Poisson regression with robust error variance was used to adjust for confounding factors for the primary outcome. Adjusted relative risk (aRR) with 95% CIs was calculated.

**Results**: Over the 5‐year study period, 166 individuals met inclusion criteria (97 pre‐SB8 vs 69 post‐SB8). There were no differences in baseline characteristics pre‐ vs post‐SB8 (Table 1). Prior to SB8, 49% of individuals opted for immediate delivery. No individuals in the post‐SB8 cohort were induced with previable ROM as the sole indication for delivery. There was a higher rate of the composite adverse outcome (20.6% vs 37.7%; aRR 2.02, 95% CI 1.2‐3.3), after adjusting for age, race and ethnicity, and gestational age at delivery. Time from rupture to delivery was longer in the post‐SB8 cohort (median [IQR]: 1 [1‐3] days vs 6 [2‐12] days, p< 0.001) and demonstrated a higher incidence of sepsis (6.2% vs 31.9%; aRR 5.15, 95% CI 2.27‐11.7).

**Conclusion**: Prior to SB8, nearly one‐half of individuals opted for pregnancy termination due to previable PROM. After SB8, risks of serious adverse outcomes increased nearly 2X, and the risk of sepsis increased over 4X. 

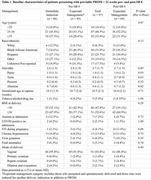

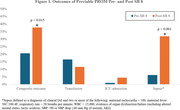

## 3 | Fetal Fraction Amplification Enables Accurate Prenatal Cell‐free DNA Screening at 8 Weeks Gestation

Lorraine Dugoff^1^; Summer Pierson^2^; Jhett Bordwell^3^; Brent Mabey^2^; Arielle B. Dorch^3^; Alexander Gutin^2^; Dmitry Pruss^2^; Diana Iliev^2^; Dale Muzzey^2^; Katie Johansen Taber^4^

*^1^University of Pennsylvania, Philadelphia, PA; ^2^Myriad Genetics, Inc., Salt Lake City, UT; ^3^Myriad Genetics, Inc., South San Francisco, CA; ^4^Myriad Genetics, Inc., South San Fransisco, CA*

8:30 AM ‐ 8:45 AM

**Objective**: Cell‐free DNA (cfDNA) screening for fetal aneuploidy is typically offered beginning at 10 weeks’ gestation. Prior to 10 weeks the proportion of placental cfDNA, known as the fetal fraction (FF), is often too low to allow for confident analysis. FF amplification, the preferential enrichment of placental cfDNA based on its shorter fragment size, may sufficiently increase FF to enable screening earlier than 10 weeks with high confidence and a low failure rate. This study aimed to evaluate the feasibility of cfDNA screening using FF amplification prior to 10 weeks’ gestation.

**Study Design**: Blood samples were collected from pregnant patients with singleton gestations at 6w0d‐9w6d and ≥10w0d. Samples were processed using an approach that incorporates FF amplification. Logistic regression was used to evaluate the proportion of samples with low FF (< 4%) as a function of gestational age (GA). Aneuploidy and sex‐call concordance were evaluated.

**Results**: 459 patients completed both blood draws. Additional cohort characteristics are described in the Table. FF ranged from 2.1‐ 34.7%, with lower GAs corresponding to lower FFs (Figure). Prior to the incorporation of FF amplification, 4.78% of samples at 10w0d had a low FF (< 4%). FF amplification allowed for comparable performance at 7w3d (the earliest date where < 4.78% of samples had FF < 4%). To reflect practical application in a clinical setting, we selected 8w0d through 9w6d as the “early GA” period. Among the 239 samples in the early GA period, one sample failed because of insufficient FF (FF = 2.5%), yielding a no‐call rate of 0.42%. Three trisomies (two T21 and one T7) were identified in the early GA period, and each was concordant with the corresponding ≥10w0d call. All 238 passing samples had concordant sex calls in the early GA and ≥10w0d GA periods.

**Conclusion**: CfDNA screening using FF amplification provided accurate results as early as 8 weeks GA with a low no‐call rate in this study population. Earlier prediction of risk for chromosomal abnormalities may provide patients with the option to have diagnostic testing with CVS at 10 weeks’ gestation.

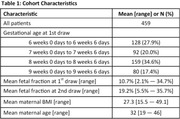

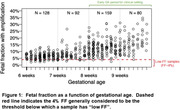

## 4 | AI Significantly Improves Detection of Prenatal Ultrasounds Suspicious for Major Congenital Heart Defects by OBGYN/MFMs

Jennifer Lam‐Rachlin^1^; Rajesh Punn^2^; Sarina K. Behera^3^; Miwa Geiger^1^; Matthias Lachaud^4^; Nadine David^5^; Sara Garmel^6^; Matthew K. Janssen^7^; Kendra Sylvester^8^; John Kennedy^9^; Jessica Spiegelman^1^; Mia A. Heiligenstein^10^; Nathan S. Fox^1^; Andrei Rebarber^1^; Greggory R. DeVore^11^; Carolyn M. Zelop^12^; Roger Bessis^13^; Marilyne Levy^14^; Bertrand Stos^14^; Malo De Boisredon^15^; Eric Askinazi^15^; Valentin Thorey^15^; Christophe Gardella^15^; Alisa Arunamata^2^

*^1^Icahn School of Medicine at Mount Sinai Hospital, New York, NY; ^2^Pediatrics ‐ Cardiology, Stanford University School of Medicine, Stanford, CA; ^3^Palo Alto Medical Foundation, Sutter Health, Palo Alto, CA; ^4^University of Grenoble Alpes, Grenoble, Rhone‐Alpes; ^5^Medical Training Center, Rouen, Haute‐Normandie; ^6^Michigan Perinatal Associates and Corewell Health, Dearborn, MI; ^7^University of Pennsylvania, Pittsburgh, PA; ^8^Perinatal Specialists of the Palm Beaches, West Palm Beach, FL; ^9^Wayne State University School of Medicine, Detroit, MI; ^10^Mount Sinai West, Astoria, NY; ^11^The Fetal Diagnostic Center of Pasadena, Pasadena, CA; ^12^Valley Health System, Paramus, NJ; ^13^Centre d'Echographie de l'Odéon, Paris, Ile‐de‐France; ^14^UE3C ‐ Unité d'explorations cardiologiques ‐ Cardiopathies Congénitales, Paris, Ile‐de‐France; ^15^BrightHeart, Paris, Ile‐de‐France*

8:45 AM ‐ 9:00 AM

**Objective**: Congenital heart defects (CHDs) are a leading cause of infant morbidity and mortality partly due to low prenatal detection rates. We evaluated whether an artificial intelligence (AI) system can improve the detection of CHDs on fetal ultrasound exams among both general OBGYNs and MFM specialists.

**Study Design**: The AI system analyzes all grayscale 2D ultrasound cines of an exam and detects 8 morphological findings associated with severe CHDs. The presence of any such finding should justify patient referral for further examination. The AI system identifies each finding as present, absent or inconclusive, and highlights frames where findings can be assessed.

A dataset of 200 ultrasound exams from 11 centers in 2 countries was collected (single pregnancy, obstetric or detailed anatomic ultrasounds, or fetal echocardiograms, at 18‐24 weeks gestation), with 100 exams having at least one suspicious finding. The ground truth for presence or absence of each finding was determined by a panel of expert fetal cardiologists. These exams were not used for the training of the AI system.

Fourteen physicians (OBGYNs and MFMs, 1‐30+ years’ experience) reviewed each exam both aided and unaided by the AI system, in randomized order, and annotated them for the presence or absence of any such finding and of each individual finding, along with confidence scores. Receiver operator characteristics (ROC) area under the curve (AUC), sensitivity and specificity were computed by comparing reviews to the ground truth.

**Results**: ROC AUC for detection of any finding was significantly higher for aided than unaided reviews: 0.97 (95% CI 0.96‐0.99) vs 0.83 (0.74‐0.91), p = 0.002 (DBM‐OR method). Similar results held for sensitivity: 0.94 (0.89‐0.98) aided vs 0.78 (0.69‐0.88) unaided and specificity: 0.97 (0.95‐0.99) aided vs 0.76 (0.63‐0.89) unaided. Mean reading time was shorter for aided (226 ± 218 s) than unaided (274 ± 241 s) reviews (p < 0.001).

**Conclusion**: Assistance by the AI system significantly improved detection of studies suspicious for CHD by OBGYNS and MFMs. AI may play a pivotal role in improving prenatal detection of CHD.

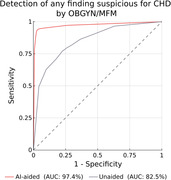

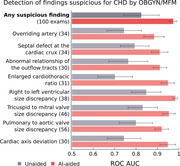

## 5 | Atosiban Versus Placebo in Threatened Preterm Birth (APOSTEL 8‐study): an International Randomized Controlled Trial

Larissa I. van der Windt^1^; Job Klumper^1^; Ruben G. Duijnhoven^1^; Marjolein Kok^1^; Ben W. Mol^2^; Kate F. Walker^3^; Fionnuala M. M. McAuliffe^4^; Joris A. M. van der Post^5^; Carolien Roos^1^; Martijn A. Oudijk^1^

*^1^Amsterdam UMC, location University of Amsterdam, Amsterdam, Noord‐Holland; ^2^Monash University, Clayton, Victoria; ^3^Centre of Perinatal Research University of Nottingham, Queen's Medical Centre, Dublin, England; ^4^UCD Perinatal Research Centre, University College Dublin, Dublin 2, Dublin; ^5^Amsterdam UMC, location University of Amsterdam, Amsterdam, Noord‐Brabant*

9:00 AM ‐ 9:15 AM

**Objective**: In many countries, threatened preterm birth (PTB) is treated with tocolytics to allow for a complete antenatal course of corticosteroids. Although effective in delaying birth, a positive effect on neonatal outcome has never been demonstrated. We assessed the effectiveness of atosiban for women with threatened PTB between 30 and 34 weeks of gestation in improving neonatal outcome.

**Study Design**: We performed a randomized double‐blind placebo‐controlled superiority trial in 26 hospitals in the Netherlands, England and Ireland. After informed consent, women with a singleton or twin pregnancy with threatened PTB between 30 and 34 weeks gestation were randomized (stratified by centre, 1:1 ratio) to intravenous atosiban or placebo for 48 hours. Primary outcome was a neonatal composite of perinatal mortality and morbidity. Treatment effect was expressed as relative risk (RR) with 95% confidence intervals (CI). To detect a reduction from 12% adverse perinatal outcome in the placebo group to 6% in the atosiban group with a power of  80%, we needed to randomise 722 women. Assuming a 5% drop‐out rate, the sample size was calculated at 760 participants. Analysis was by intention‐to‐treat principle.

**Results**: From December 2017 to July 2023, we randomized 755 participants to atosiban (n = 377) or placebo (n = 378). Primary outcome was available for 752 participants with 884 infants. The primary outcome occurred in 37/449 (8.2%) infants in the atosiban group and 40/435 (9.2%) in the placebo group (RR 0.90, 95% CI 0.58 to 1.40). There were two (0.4%) and four (0.9%) perinatal deaths respectively (RR 0.48, 95% CI 0.09 to 2.63). There were no significant differences in other neonatal outcomes. Prolongation of pregnancy > 48 hours was significantly more often achieved in the atosiban group (292/375, 77.5%) compared to placebo (261/377, 69.2%) (RR 1.13, 95% CI 1.03 to 1.24).

**Conclusion**: We did not demonstrate superiority of atosiban over placebo in improving neonatal outcomes as a treatment for threatened PTB between 30 and 34 weeks of gestation. This questions the widespread use of tocolytics in many countries.

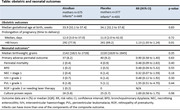

## 6 | Prediction of Severely Small‐for‐Gestational‐Age Infants Using a Novel Cell‐free RNA Model

Morten Rasmussen^1^; Kara M. Rood^2^; Aram Saravani^1^; Michal A. Elovitz^3^; Carrie Haverty^4^; Alison Moe^1^; Alison Cowan^1^; Arkady Khodursky^1^; Maneesh Jain^1^; Manfred Lee^1^; Thomas F. McElrath^5^; Elizabeth F. Sutton^6^; Arun Jeyabalan^7^; George R. Saade^8^; Antonio F. Saad^9^; Luis D. Pacheco^10^; Joseph R. Biggio, Jr^11^; Ebony B. Carter^12^; Antonina I. Frolova^13^; Esther Park‐Hwang^14^; Cynthia Gyamfi‐Bannerman^15^; Ai‐ris Y. Collier^16^; William A. Grobman^2^; Vincenzo Berghella^17^

*^1^Mirvie Inc., South San Francisco, CA; ^2^The Ohio State University, Columbus, OH; ^3^Icahn School of Medicine at Mount Sinai, New York, NY; ^4^Mirvie Inc., South San Fransisco, CA; ^5^Brigham Women's Hospital, Boston, MA; ^6^Woman's Hospital, Baton Rouge, LA; ^7^University of Pittsburgh School of Medicine, Pittsburgh, PA; ^8^Macon & Joan Brock Virginia Health Sciences Eastern Virginia Medical School at Old Dominion University, Norfolk, VA; ^9^Inova Health, Falls Church, VA; ^10^University of Texas Medical Branch, Galveston, TX; ^11^Ochsner Health, New Orleans, LA; ^12^University of North Carolina, Chapel Hill, NC; ^13^Washington University School of Medicine, St. Louis, MO; ^14^Multicare, Orlando, FL; ^15^University of California, San Diego, San Diego, CA; ^16^Beth Israel Deaconess Medical Center, Boston, MA; ^17^Sidney Kimmel Medical College of Thomas Jefferson University, Philadelphia, PA*

9:15 AM ‐ 9:30 AM

**Objective**: Pregnancies  with suspected severe fetal growth restriction (FGR) require increased surveillance and earlier delivery due to their associated risk of stillbirth and other comorbidities. However, many  infants born at < 3^rd^ percentile (SGA3) are not detected until after delivery. We sought to understand the extent to which SGA3 can be predicted with a novel cell‐free RNA (cfRNA) model, compared to the antepartum detection rate of SGA3.

**Study Design**: From a prospective cohort, 5,195 subjects with singleton gestations were enrolled from 11 U.S. sites and direct‐to‐participant mobile recruitment. Maternal blood was drawn between 17w4d‐22w0d and pregnancy outcomes were adjudicated. Growth ultrasounds were performed for usual clinical indications. SGA3 was based on the Fenton growth chart. A cfRNA model was developed with features selected using sure independence screening and fit using cross‐validation and L1 to reduce feature count. Rates of antepartum detection of SGA were estimated via the rate of FGR diagnosis.

**Results**: 115 (2.2%) delivered SGA3 infants, of whom 44.3% (N = 51) were detected antenatally with FGR. SGA3 was associated with preeclampsia, nulliparity, younger age, and Black race. A cfRNA model driven by *COL24A1* for prediction of SGA3 demonstrated an AUC of 0.75 (Fig). Excluding subjects with preeclampsia (N = 624, 22 SGA3) demonstrated comparable performance (AUC = 0.75), as did exclusion of subjects with chronic HTN and T1/T2DM (N = 555, 12 SGA3; AUC = 0.76). The cfRNA model predicted 59.1% (N = 68) of SGA3 infants. 30.4% (N = 35) were predicted to be SGA by cfRNA modeling but not FGR diagnosis vs. 14.8% (N = 17) predicted by FGR diagnosis but not cfRNA.

**Conclusion**: This large diverse study shows that antenatal detection of FGR is lacking, with the majority of SGA3 infants undiagnosed prior to birth. Improved diagnostic tools are required to identify these high‐risk pregnancies. A cfRNA model identified the majority of SGA3 infants, independent of preeclampsia status. This novel cfRNA model represents a promising step toward the improved antepartum prediction and detection of severe FGR.

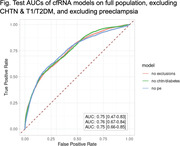

## 7 | Metformin Paradoxically Increases Insulin Resistance During Pregnancy in a Rhesus Macaque Model

Enrico R. Barrozo^1^; Tyler Dean^2^; Claire E. Jensen^3^; Melissa A. Suter^1^; Maxim D. Seferovic^1^; Kristin Sauter^2^; Jacob E. Friedman^4^; Maureen Gannon^5^; Stephanie R. Wesoloski^6^; Carrie E. McCurdy^7^; Paul Kievit^2^; Kjersti M. Aagaard^8^

*^1^Baylor College of Medicine and Texas Children's Hospital, Houston, TX; ^2^Oregon National Primate Research Center, Beaverton, OR; ^3^University of North Carolina, Chapel Hill, NC; ^4^Harold Hamm Diabetes Center, University of Oklahoma Health Sciences Center, Oklahoma City, OK; ^5^Vanderbilt University Medical Center, Nashville, TN; ^6^University of Colorado, Anschutz Medical Campus, Aurora, CO; ^7^University of Oregon, Eugene, OR; ^8^Boston Children's Hospital, Division of Fetal Medicine and Surgery, Boston, MA; HCA Healthcare and HCA Healthcare Research Institute, Nashville, TN; HCA Texas Maternal Fetal Medicine, Houston, TX; Baylor College of Medicine and Texas Children's Hospital, Houston, TX, Boston, MA*

9:30 AM ‐ 9:45 AM

**Objective**: Normal human and non‐human primate pregnancy physiology includes mild insulin resistance, which enables appropriate fetal growth & species‐specific accumulation of fetal adiposity. With rising diabetes in pregnancy, metformin use has become common despite its inferiority to insulin in managing maternal hyperglycemia. We hypothesized metformin inferiority during pregnancy may be due to differing pathophysiology of insulin resistance in pregnancy and tested this hypothesis in our recent Rhesus macaque model.

**Study Design**: Dams (n = 75) were randomized to vehicle control (VEH) or metformin (MET, 10 mg/kg twice daily) at pregnancy confirmation by GD30 and allocated to control (CHOW) or an isocaloric 35% fat Western‐style diet (WSD). Glucose tolerance tests were conducted pre‐pregnancy (Baseline), 3rd trimester, and 3 months postpartum. AUC for glucose and insulin, HOMA‐IR, QUICKI, and Matsuda Index were calculated as insulin resistance (IR) and sensitivity (IS) measures, respectively. Data were analyzed using two‐way ANOVA or mixed‐effects models and Tukey's test for multiple comparisons (p< 0.05).

**Results**: Consistent with pregnancy physiology, dams were insulin resistant during pregnancy (insulin AUC p< 0.0001) but not hyperglycemic (p = 0.3; Fig 1). Relative to VEH, initiation of metformin by GD30 resulted in increased IR (HOMA‐IR, p = 0.03) and decreased IS (QUICKI & Matsuda, p = 0.001 & p< 0.001), both of which appropriately varied during gestation. Metformin was ineffective at treating IR during pregnancy and postpartum, with a paradoxical effect of significantly increased IR with metformin use compared to VEH (baseline vs 3rd trimester q< 0.05, Fig 2). Results were consistent when stratified by fetal sex and lactation status.

**Conclusion**: In our non‐diabetic rhesus macaque model, we found that the pathophysiology and metformin‐responsiveness of maternal pregnancy‐related IR differ from non‐pregnancy IR. These data are consistent with the lack of efficacy in human trials and underscore the need for longitudinal studies on the mechanisms and long‐term health risks of metformin exposure during pregnancy.

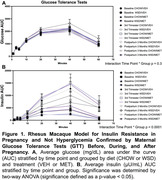

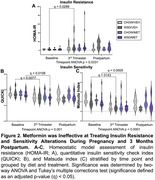

## 8 | INTER‐ACT Interpregnancy and Pregnancy Lifestyle Intervention for a Healthy Future: a Randomized Controlled Trial

Yael Winter Shafran^1^; Hanne Van Uytsel^2^; Annick Bogaerts^2^; Lieveke Ameye^2^; Roland Devlieger^3^

*^1^Kaplan Medical Center and REALIFE Research Group, Rehovot, HaMerkaz; ^2^REALIFE Research Group, KU Leuven, Leuven, Vlaams‐Brabant; ^3^University Hospital Leuven, Leuven, Vlaams‐Brabant*

9:45 AM ‐ 10:00 AM

**Objective**: Maternal pre‐pregnancy BMI and gestational weight gain (GWG) influence pregnancy outcomes. Interventions during pregnancy show limited success, highlighting the need for pre‐pregnancy interventions. This study evaluated a comprehensive lifestyle intervention from the interpregnancy to subsequent pregnancy periods in individuals with a history of excessive GWG, aiming to reduce pregnancy complications.

**Study Design**: The INTER‐ACT trial is a prospective multicenter randomized controlled trial (RCT) conducted from 2017 to 2019. A total of 1,450 participants with were randomized, with 390 experiencing a singleton pregnancy. The intervention included face‐to‐face coaching and an E‐Health app focusing on nutrition, physical activity, and mental well‐being. The control group had measurements and surveys. The primary outcome was a composite of pregnancy‐induced hypertension, gestational diabetes, cesarean section, and large‐for‐gestational‐age infants.

**Results**: The lifestyle intervention did not significantly reduce the composite outcome overall (OR 1.29, 95% CI 0.85 to 1.96). Adherence dropped substantially during the following pregnancy, with only 55% attending at least one coaching session and 38% using the app. A subanalysis revealed that participants who adhered to either the intervention or control follow‐up had a significantly reduced composite outcome compared to non‐adherent participants, with ORs of 0.50 (95% CI: 0.26 to 0.98) and 0.48 (95% CI: 0.26 to 0.90), respectively.

**Conclusion**: This innovative, inclusive large‐scale RCT highlights the potential of lifestyle interventions to improve pregnancy outcomes and underscores the importance of adherence and motivation. While the INTER‐ACT intervention did not lower the composite outcome rate, those who maintained adherence either to the intervention or the follow‐up showed significant benefits. Future trials should explore the impact of lifestyle interventions across diverse BMI ranges, particularly in the preconception and interpregnancy periods, and examine how to motivate participants to take a more active and aware role in their lifestyle choices.